# Action-FRET of a Gaseous Protein

**DOI:** 10.1007/s13361-016-1449-2

**Published:** 2016-08-09

**Authors:** Steven Daly, Geoffrey Knight, Mohamed Abdul Halim, Alexander Kulesza, Chang Min Choi, Fabien Chirot, Luke MacAleese, Rodolphe Antoine, Philippe Dugourd

**Affiliations:** 1 Institut Lumière Matière, Université Lyon 1-CNRS, Université de Lyon, 69622 Villeurbanne cedex, France; 2 Institut des Sciences Analytiques, Université Lyon 1 – CNRS, Université de Lyon, 69622 Villeurbanne Cedex, France

**Keywords:** FRET, Ubiquitin, Molecular dynamics, Action FRET

## Abstract

Mass spectrometry is an extremely powerful technique for analysis of biological molecules, in particular proteins. One aspect that has been contentious is how much native solution-phase structure is preserved upon transposition to the gas phase by soft ionization methods such as electrospray ionization. To address this question—and thus further develop mass spectrometry as a tool for structural biology—structure-sensitive techniques must be developed to probe the gas-phase conformations of proteins. Here, we report Förster resonance energy transfer (FRET) measurements on a ubiquitin mutant using specific photofragmentation as a reporter of the FRET efficiency. The FRET data is interpreted in the context of circular dichroism, molecular dynamics simulation, and ion mobility data. Both the dependence of the FRET efficiency on the charge state—where a systematic decrease is observed—and on methanol concentration are considered. In the latter case, a decrease in FRET efficiency with methanol concentration is taken as evidence that the conformational ensemble of gaseous protein cations retains a memory of the solution phase conformational ensemble upon electrospray ionization.

**Electronic supplementary material:**

The online version of this article (doi:10.1007/s13361-016-1449-2) contains supplementary material, which is available to authorized users.

**Graphical Abstract:**
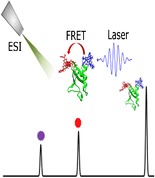
ᅟ

## Introduction

In recent years there has been an increasing interest in the study of biological molecules in the gas phase using mass spectrometry techniques [1]. Such experiments are attractive since the solvent-free environment of the gas phase allows the study of the intramolecular interactions that play a significant role in protein folding, and the intermolecular interactions leading to the formation of protein complexes. The mass spectrometer allows the isolation of complexes of specific sizes and charge, providing a simple technique by which the various stages of protein aggregation can be probed [2, 3]. Coupled to this, it has been shown that the mild ionization conditions prevalent in electrospray ionization (ESI) can successfully transport intact such large molecular frameworks and retain solution-phase conformational ensembles into the gas phase [2, 4]. One key open question is the exact nature of the relationship between the structural ensembles present in the solution- and gas phases, and how does the solution-phase structural ensemble evolve when moved to the gas phase. This question is fundamental for the integration of gas-phase techniques to study protein structure and function into the broad topic of structural biology.

To this end, it is important to transpose from solution to gas phase some typical protein biophysical characterization techniques. Current gas-phase methods have no direct correlates in the solution phase, and hence only indirect comparisons can be drawn between disparate measures of protein structure and function in the two media. One solution-phase technique that is commonly used to study biomolecular systems is Förster resonance energy transfer (FRET) [5, 6]. FRET relies on the energy transfer between two chromophores, termed the donor and the acceptor, where the fluorescence spectrum of the donor has a large overlap with the absorption spectrum of the acceptor; the high sensitivity to the distance between the chromophores that displays an R^−6^ dependence (i.e., the so-called “molecular ruler” [7]).

The transposition of FRET to the gas phase has been demonstrated by several groups via the detection of fluorescence or fragmentation, with a concurrent development of computational methods for obtaining theoretical FRET efficiencies from chromophore-tagged structural ensembles [8–15]. This emerging technique has been recently applied to biologically relevant systems to elucidate differences in the structural landscape of mutants of the 12–28 fragment of amyloid-β in order to probe differences in aggregation phenomena, and has also been applied to probe the structure of dimers of the same system, showing the coexistence of two distinct binding motifs [14, 16].

The next developmental step for gas-phase FRET techniques is to show that it is possible to measure the FRET efficiency of the gas-phase structural ensemble of a doubly tagged protein. Czar et al. recently presented the first example of gas-phase FRET on GB1,the immunoglobulin G-binding domain of protein G, which is known to retain its secondary structure across a large range of pH values in solution, with the aim of finding how much structure is preserved when removal of stabilizing solvent interactions are removed. Changes in the fluorescence spectra and lifetimes of doubly tagged GB1 as a function of the charge state and finding a Coulomb-driven unfolding as the charge state increased, and also indicated the presence of multiple conformations for the 5+ charge state [17]. The work of Czar et al. expands the use of gas-phase FRET to an intact protein, and continues the current increasing use of FRET to study the structure of gas-phase peptides and proteins [18].

In addition to fluorescence-based techniques, detection of photofragments as a FRET reporter uses the discerning advantage of mass spectrometry to provide a methodology for FRET measurements that is highly sensitive and which we call action-FRET. Here, we present the first action-FRET measurements performed on a double cysteine mutant of ubiquitin. Ubiquitin presents an ideal system for an initial gas-phase FRET study since it has been examined previously in great detail in vacuo, with studies into the influence of charge state, initial solution conditions, and trapping time on the conformational profiles [19–30].

## Experimental

### Methods

**Figure 1:**
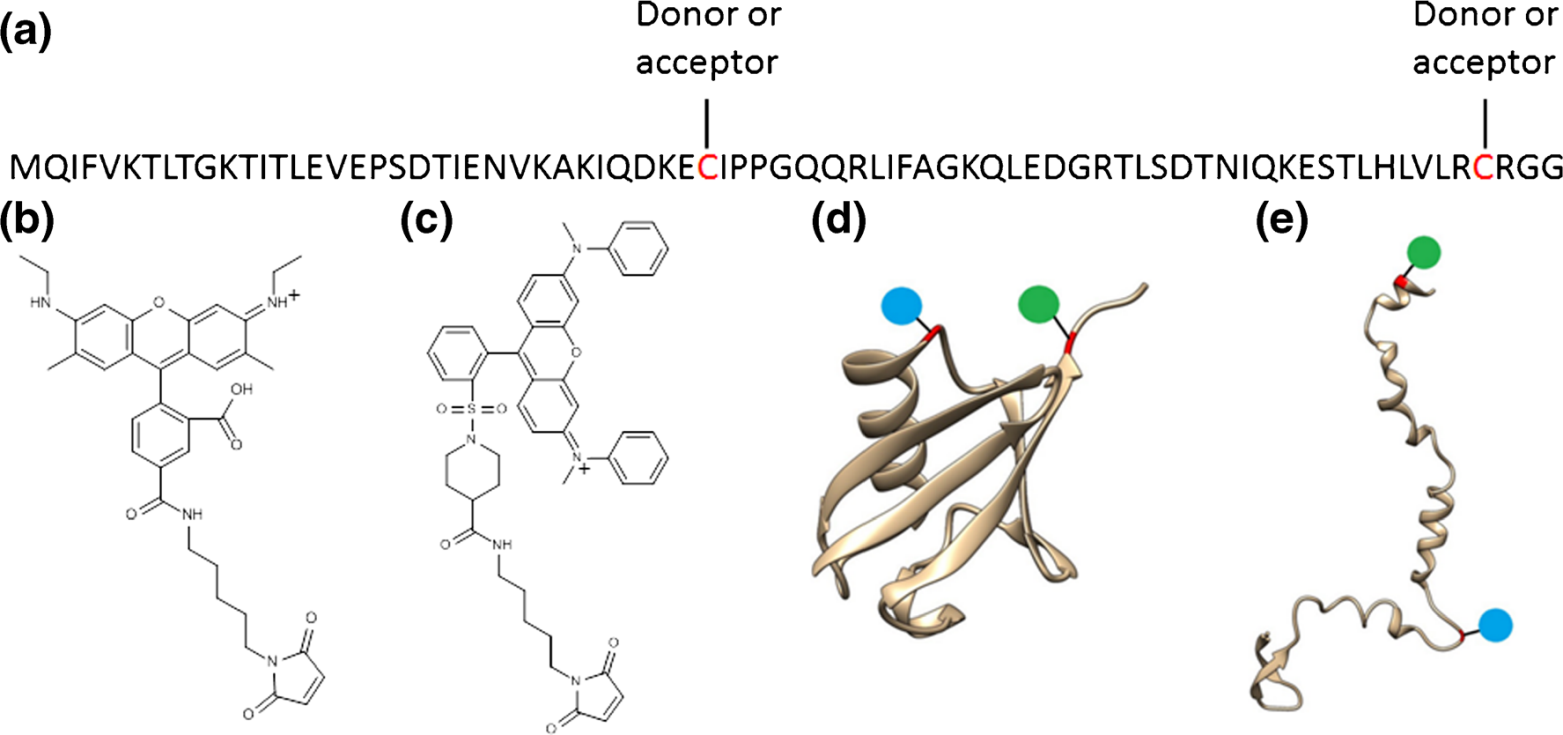
**(a)** Sequence of the double cysteine ubiquitin mutant with grafting. **(b)** The structure of the donor chromophore rhodamine 575-C_5_-maleimide. **(c)** The structure of the acceptor chromophore QSY7-C_5_-maleimide. **(d)** Secondary structure of the native state of ubiquitin (pdb file 1UBQ) with chromophore position indicated by blue and green circles. **(e)** A representation of the secondary structure of the A-state of ubiquitin taken from an MD simulation (see text), with chromophore positions indicated by blue and green circles

Rhodamine 575 C_5_-maleimide (Setareh Biotech, Eugene, OR, USA) and QSY 7 C_5_-maleimide (Invitrogen, Carlsbad, CA, USA) were dissolved in DMSO (1 mg/100 μL), and an equimolar (100 μM) solution of both chromophores prepared in H_2_O. Bovine ubiquitin (Sigma Aldrich, St. Louis, MO, USA) was diluted in H_2_O, 1:1 H_2_O:CH_3_OH or CH_3_OH with 1% acetic acid by volume to a final concentration of 10 μM; 10 mg of G35C L73C ubiquitin mutant (C-UBI-C, see Figure 1a) was purchased (Genscript, Piscataway, NJ, USA) and dissolved in phosphate buffered saline solution for storage (1 mL, 0.37 mg/mL). Prior to grafting of the chromophores, each 1 mL aliquot was treated with an excess of TCEP (28 mM in H_2_O) to remove any disulphide bonding, and subsequently purified by HPLC (Agilent, Santa Clara, CA, USA) using a C4 column (Poroshell, 4.6 × 150 mm, Analytical) employing a H_2_O 0.1% trifluoroacetic acid to CH_3_CN 0.1% trifluoroacetic acid gradient. The collected sample was dried under a constant flow of N_2_ gas, rehydrated in 500 μL H_2_O, and neutralized by dropwise addition of 1% NH_4_OH in H_2_O. To the purified, neutralized ubiquitin solution was added 110 μL of the 100 μM chromophore solution, and the resulting solution was left for 24 h at room temperature to ensure completion of the tagging reaction. For use in electrospray ionization, the reaction solutions were diluted to a concentration of 10 μM in either H_2_O, 1:1 H_2_O:CH_3_OH or CH_3_OH with 1% acetic acid by volume.

The action-spectroscopy experimental setup has been described in detail previously [31], and a brief description is given here. A dual linear ion trap mass spectrometer (LTQ Velos, Thermo Fisher Scientific, San Jose, CA, USA) was modified by positioning a fused silica window (3 mm thick, 1 inch diameter) at the back end of the instrument, and 1–2 mm diameter circular openings in the trapping electrodes to allow coupling of light source and trapped ions. In order to optimize laser transmission, the central hole of the electrode closest to the fused silica window was enlarged to 5 mm in diameter. The light source used is a Panther Ex OPO pumped by the third harmonic of a Surelite II Nd:YAG laser (Continuum, Santa Clara, CA, USA). A repetition rate of 10 Hz and pulse widths of 5 ns were used. The beam was focused into the first ion trap using a 1000 mm focal length convergent lens. A mechanical shutter, synchronized with the mass spectrometer, was used to stop the beam at all times except the “ion activation window,” that is, the time after ion accumulation and before the mass analysis. A single laser pulse was used for the irradiation of the trapped ions, and when irradiating ions, the normalized collision energy is kept at zero.

A detailed description of the action-FRET methodology and implementation can be found in detail elsewhere, and will be discussed in more detail below [14, 31]. Briefly, each charge state was isolated and irradiated by one laser pulse of either 505 or 545 nm light, corresponding to the absorption maximum of the donor and the acceptor chromophore, respectively, (which undergo only minimal shifts upon covalent attachment). At each wavelength, the resulting mass spectrum following irradiation was averaged for a period of 2 min. The laser power was measured immediately preceding and following the accumulation of a mass spectrum for a period of 1 min, and the average of these two power readings was taken as the laser power for intervening mass spectrum. The relative intensity of the fragments specific to photo-excitation of the acceptor chromophore was determined for each mass spectrum and normalized to the laser fluence (power × wavelength) [12]. The FRET efficiency was then determined by taking the normalized relative intensity at 505 nm and dividing it by the normalized relative intensity at 545 nm for each pair of measurements. The final value was taken as the average of these FRET efficiency values, whilst their standard deviation was taken as the error. A correction of −0.25 is applied to all the final FRET efficiency values to account for the non-zero fragmentation efficiency of QSY7 at 505 nm [14].

Ion mobility measurements were performed on a tandem drift tube spectrometer described in detail elsewhere [32]. Here, ions were analyzed using 4 Torr helium as a buffer gas at room temperature and utilizing drift voltages between 250 and 500 V. Collision cross section (CCS) values were determined by measuring ion arrival times as a function of the inverse voltage value across second drift tube, and all reported CCS values and profiles were obtained using helium.

Circular dichroism experiments were performed on a ChiraScan qCD spectrometer (Applied Photophysics, Leatherhead, UK). Concentrations of approximately 100 μM were used when obtaining circular dichroism spectra.

### Computational

The structure of chromophore-tagged ubiquitin was studied by molecular dynamics (MD) simulations in the frame of an Amber99/GAFF force field parametrization [33, 34], which has been successfully applied to determine structural properties of chromophore-tagged amyloid-β monomers and dimer in the gas phase [14, 15]. The same parameter set is used both for gas-phase and preparative solution-phase simulations.

Topology manipulations and the generation of GAFF topologies were performed using the acpype [35] interface of the antechamber tool within ambertools14 [36]. Molecular dynamics simulations were performed using the Gromacs 5.1 [37, 38] engine with the velocity verlet integrator. Preparative solution-phase MD was performed using a pre-equilibrated water–methanol box. This box was created from adding 2250 (TIP3P) water molecules and 1000 (GAFF) CH_3_OH molecules (roughly corresponding to a 1:1 vol mixture) into an orthorhombic box of 4.5 nm edge-length with the PACKMOL software [39]. After several optimization and NVT equilibration (2.5 ns, Nosé-Hoover [40] temperature coupling, T = 250 K, τ = 0.1 ps, particle mesh Ewald electrostatics treatment [41] with 1 nm Coulomb cutoff) cycles with slightly increasing box size, the pressure dropped to an acceptable value (below 100 bar) so that the NPT equilibration (Parinello-Rahman pressure coupling [42], p = 1 bar, τ = 10 ps) converged quickly to a density of 943 kg m^−3^. The equilibration procedures have also been used with different temperatures for the ubiquitin-containing simulations as indicated in the main text. NPT production runs (setup as before), employ bond-constraints using the LINCS algorithm [43] and an increased integration time step of 2.5 fs.

The chromophores (rhodamine 575 C_5_-maleimide and QSY7 C_5_-maleimide functionalized cysteine residues) have been treated within a GAFF parametrization as described previously and the details of the FRET-efficiency calculations used herein are also described in detail elsewhere [15]. In brief, the FRET efficiencies are calculated from chromophore separations and orientation factors of the ensemble of structures found in the molecular dynamics runs and using spectral parameters of the chromophores from experiments and TDDFT calculations (see Ref. [15], R_0_ = 40.3 Å with chromophores being isotopically oriented). Gas-phase MD simulations were performed after removing all solvent and ions in the box, using a 1 fs time step and neither cut-offs for the evaluation of non-bonded interactions nor bond-constraints.

## Results and Discussion

### Chromophore Tagging

In order to perform gas-phase FRET experiments on proteins, it is of vital importance that the tagging protocol is performed under mild conditions in order to minimize the risk of perturbing the native protein structure in solution. Equally, it is important to demonstrate that the structure of the protein is unlikely to be drastically altered by the cysteine point mutations required for producing a doubly tagged mutant. The choice of mutation site is also constrained by the requirement that the two residues undergo a large change in separation upon the conformational change that is being probed. In this case, the conformational change that is to be probed is the unfolding of native (N) ubiquitin to the A-state that is reported to be the dominant structure in solutions containing acidified methanol [44, 45]. If the two structures are considered, Figure 1b and c, residues G35-Q40 and R72-G76 undergo a large increase in separation upon change in conformation. Having located two regions of the sequence to target for mutation, a single residue must be selected for mutation to cysteine (an alternative—particularly feasible at N- or C-terminus—is addition of a single cysteine residue) [14]. In the 35–40 region, the two proline residues are involved in the turn between helical and β-sheet regions and can be discarded. Similarly, residues with polar side chains are often important to secondary structure and are unsuitable candidates. Hence, G35 is chosen as the location for the first mutation site, since G→C is a relatively common, naturally occurring mutation [46]. In the 72–76 region, the two arginine residues can be discarded for the reasons given above. In addition, since the C-terminal region of ubiquitin is known to be flexible, it is desirable to avoid mutating too close to the C-terminus, eliminating the terminal glycine residues as well as a C-terminus cysteine addition [47, 48]. This leaves L73 as the only remaining residue, which is therefore chosen as the second mutation site, which can be seen as a sensible mutation since L→C is again a relatively common, naturally occurring mutation [46].

To show that the mutations described above do not cause a large change in the secondary structure of the protein, and also to show that the experimental protocol for preparing the protein sample for tagging with chromophores does not lead to non-native structures, solution phase circular dichroism measurements were performed on both C-UBI-C and bovine ubiquitin. The comparison of the circular dichroism of C-UBI-C and wild-type bovine ubiquitin in H_2_O with 1% acetic acid by volume is shown in Supplementary Figure S1. It must be noted that two separate y-axis scales are used since the concentration of C-UBI-C following HPLC was much lower than the initial 1 mg/1 mL, which was used for the bovine ubiquitin, and this leads to an overall decrease in the magnitude of the circular dichroism signal. The circular dichroism of both the wild-type bovine ubiquitin and the C-UBI-C mutant are similar. Also shown in Supplementary Figure S1 is a reference spectrum of homo sapiens ubiquitin in H_2_O as the dashed red line [49, 50]. The agreement between the reference spectrum and the two experimentally determined curves is good, indicating that there is no large change in secondary structure either by the two point mutations or by the experimental purification protocol.

It is not possible to perform the same circular dichroism measurements for tagged ubiquitin since both chromophores possess intense ultraviolet absorption features [31] and, hence, it is difficult to obtain direct information on changes in the solution structure following chromophore tagging. It may be noted that the successful use of similar chromophores in solution-phase FRET experiments indicates that such effect must be limited if any useful information is to be derived at all. In the gas phase, however, it is possible to obtain a more detailed picture of the influence of chromophore tagging either by examining changes in the charge state distributions or by ion mobility spectrometry to determine collision cross sections.

**Figure 2:**
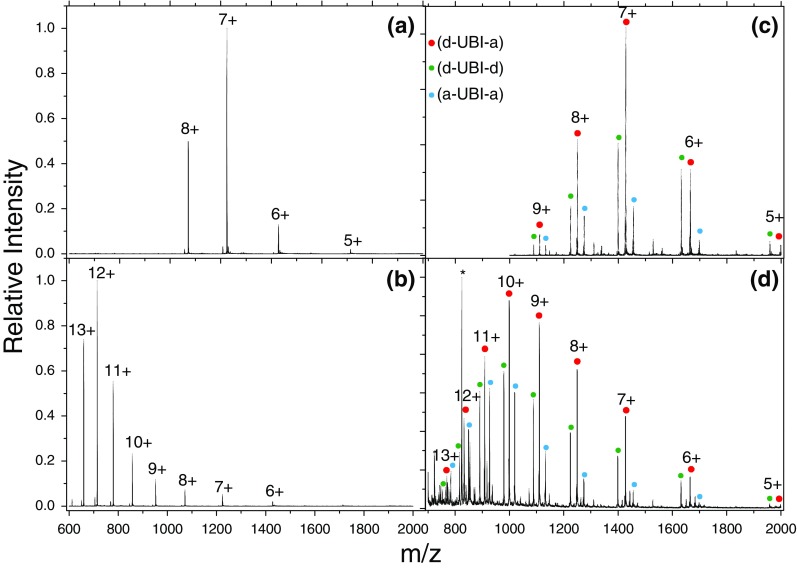
Mass spectrum following ESI of bovine ubiquitin and [d-UBI-a]^z+^ cations in H_2_O **(a)** and **(c)** and 1:1 H_2_O:CH_3_OH **(b)** and **(d)**, both with 1% acetic acid by volume. Red circles denote doubly tagged species containing both a donor **(d)** and acceptor **(a)** chromophore. Smaller green and blue circles denote tagging with two **(d)** or **(a)** chromophores, respectively. The asterisks in **(d)** denote peaks associated with unreacted acceptor chromophore, which is the cause of the congestion of peaks below *m/z* 900. The labels represent the total charge state of the system

Figure 2 show the mass spectrum of bovine ubiquitin and doubly tagged C-UBI-C in either H_2_O 1% acetic acid by volume (Figure 2a and c, respectively) or 1:1 H_2_O:CH_3_OH 1% acetic acid by volume (Figure b) and d, respectively), where the labels denote the total charge state. For wild-type bovine ubiquitin, the mild condition in Figure 2a leads to a narrow distribution of low charge states with 7+ being most intense. In the denaturing conditions, a broad range of highly charged species are observed. These changes are related to the ESI process, whereby compact native structures are charged less than unfolded denatured ones [51, 52]. Although in the native fold only a part of the residues is solvent-accessible and thus is titrable, the majority of residues in the unfolded species are solvent-accessible and thus such structures are more prone to high protonation states.

Each charge state of the doubly tagged ubiquitin in Figure 2c and d is split into a triad of peaks, attributable to the lack of specificity in the tagging reaction and corresponding to donor-donor (d-UBI-d), donor-acceptor (d-UBI-a), and acceptor-acceptor (a-UBI-a) tagged ubiquitin (green, red, and blue dots, respectively). The charge state distribution in mild conditions resembles closely that seen in Figure 2a for untagged ubiquitin, indicating that the chromophores are not causing a denaturing effect of the protein. In the acidified binary mixture of water and methanol in Figure 2d, a broader range of charge states are observed, although peaking at 10+ in the tagged ubiquitin rather than 12+ seen in untagged ubiquitin. It appears that the tagging protocol is not greatly perturbing the secondary structure in such a way as to completely denature the protein.

In order to further elucidate the influence of the chromophores, ion mobility measurements were performed on tagged and untagged C-UBI-C. To simplify the mass spectrum, only the acceptor chromophore was used, and a reaction solution was obtained by adding 50 μL of a 100 μM solution of acceptor chromophore to a 500 μL aliquot of C-UBI-C. This gives a mixture of tagged and untagged protein in the sample, allowing direct comparison of C-UBI-C and a-UBI-a under identical solution and experimental conditions. A solution in 1:1 H_2_O:CH_3_OH with 1% acetic acid by volume using this reaction solution was prepared for ESI. The resulting CCS values for the main peak in the drift time distribution for C-UBI-C and a-UBI-a as a function of the charge state are shown in Supplementary Figure S2. Charge states ranging from 5+ to 12+ were observed for C-UBI-C and 7+ to 12+ for a-UBI-a. From the CCS values, the trends between tagged and untagged proteins are very similar except that there is a shift of +2 in the charge state for the tagged species. Since the chromophores possess two permanent positive charges, a given charge state z of tagged ubiquitin will have n = z–2 protons on the protein itself. Hence, this observation of a shift of +2 comparing the CCS trend of tagged and untagged ubiquitin suggests that it is the protonation state of the protein, rather than the overall charge state, which is the dominant influence on unfolding of the protein in both cases.

It is, nevertheless, also instructive to compare the CCS profiles for tagged and untagged ubiquitin to confirm that they remain comparable (shown in Supplementary Figure S3). Again, similar unfolding behavior is observed comparing the same protein protonation states. A difference between tagged and untagged species is that there is an increased amount of extended structures for the tagged species. This can be attributed to the higher charge state in the tagged protein causing a destabilization of the protein structure compared with the same protonation state of the untagged protein. So whilst the unfolding dynamics appear to depend on the protonation state of the protein, the presence of the charged chromophore is influencing slightly the relative stability of the protein structures.

### Computational Modeling

**Figure 3:**
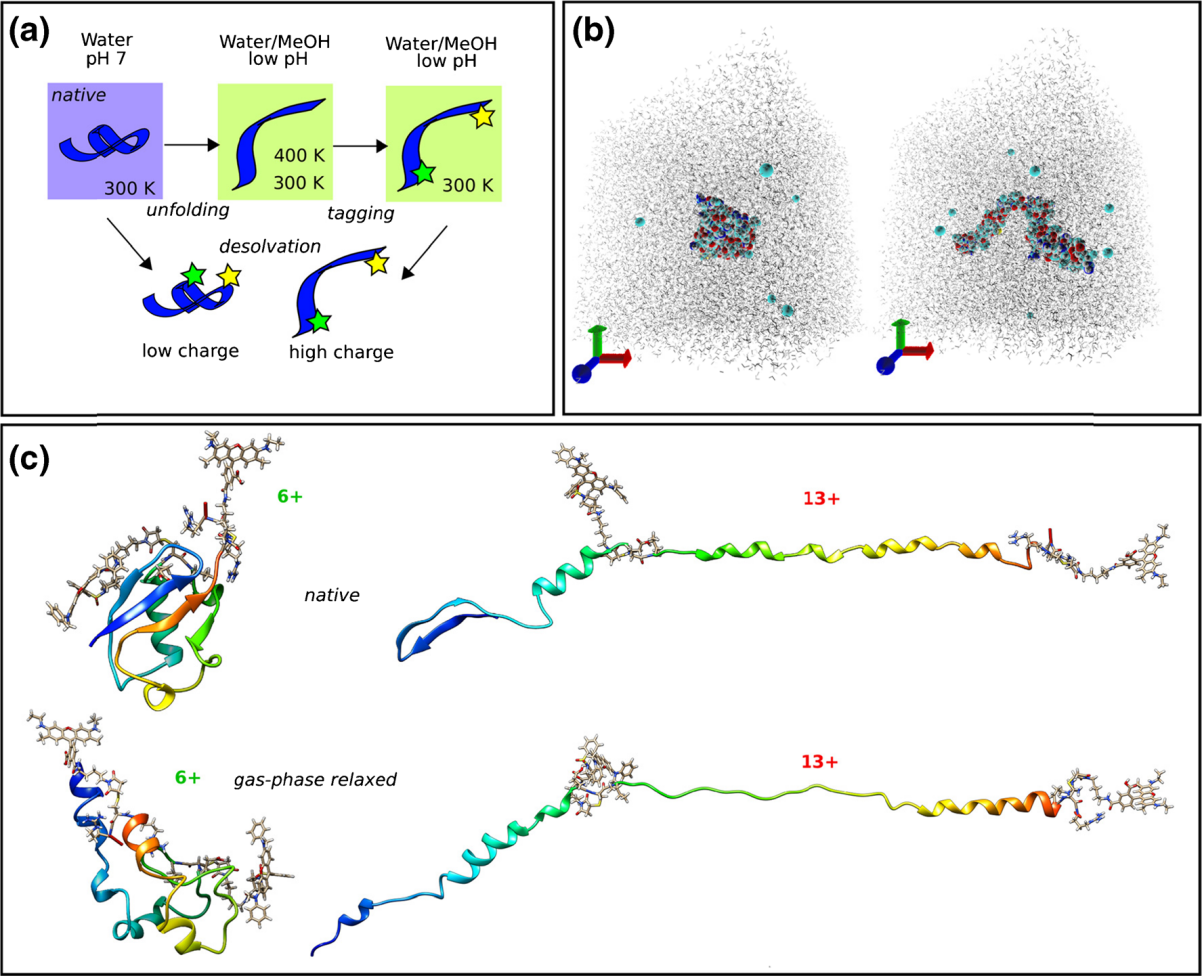
Computational strategy for modeling of chromophore-tagged ubiquitin structures in the gas phase. **(a)** Schematic overview of simulation conditions chosen for different stages (stars denote chromophores), see main text; **(b)** color-inverted graphical representation of the H_2_O/CH_3_OH simulation box (including ions) featuring a natively folded ubiquitin (compact, left) and a partially unfolded structure (elongated, right); **(c)** structural properties of chromophore-tagged ubiquitin in the gas phase in a low (6+) and a high (13+) charge state after equilibration in the gas phase and replica-exchange MD. Peptide backbone in ribbon representation color coded by residue index (blue N-terminus, red C-terminus)

In order to model the FRET efficiencies for gaseous tagged ubiquitin cations, it is imperative to have an overall robust computational strategy to obtain the structural families according to the experimental conditions. This must include analysis of the partial unfolding of both tagged and untagged ubiquitin in solution, the relaxation of these solution-phase structures upon transposition to the gas phase, and finally estimation of the FRET efficiency in the gas phase, Figure 3a. First, unfolded structures of the untagged protein must be obtained by performing MD simulation in explicit solvent according to different solution/pH conditions. The results of this procedure can be compared with experimental determinations of solution phase secondary structure, such as circular dichroism, in order to demonstrate that the computationally derived structures are physically relevant.

Wild-type ubiquitin was partially unfolded by a MD simulation in explicit water(TIP3P)/methanol(GAFF) by protonation of the protein assuming a low pH, accelerated by a variable temperature scheme to control and speed up the structural transformation [53]. The crystal structure of ubiquitin (pdb file 1UBQ) in the 12+ charge state was produced by protonation of all basic residues and neutralisation of all acid residues (see Supplementary Table S1) and after optimization charge neutrality was restored by adding 12 Cl^−^ ions. Compared to a procedure described earlier [54] to model the A-state in water, a pre-equilibrated water/methanol solvent box (see computational details) under milder conditions was used here. To accommodate the unfolding process, a box with edge-length of 10 nm (approximately 100 k atoms) was chosen. Within 43 ns of production run, partial unfolding was observed by a transition from the native compact structure to an elongated configuration (Figure 3b, right). This configuration was immediately quenched to a temperature of 300 K again and at the end of a 37.5 ns NPT simulation the partially unfolded structure model was obtained. The 400 K unfolding run was extended to 75 ns, which led to a further structural transition to an elongated structure with high helical content and a completely unfolded N-terminal region.

To validate that our structural model complies with the experimentally observed unfolded structures, circular dichroism measurements were performed for ubiquitin solvated in H_2_O, H_2_O/CH_3_OH, and CH_3_OH, each with 1% acetic acid by volume, Supplementary Figure S4a. Predicted circular dichroism for native (1 UBQ), partially and fully unfolded species described above were calculated using the DichroCalc web-interface [55], shown in Supplementary Figure S4b. Analysis of secondary structure elements reveals that the partially unfolded structure appears to possess a significant disordered character with an α-helical content ranging from 0 up to 28% (Supplementary Table S2) throughout the sample. Compared with the native state, a loss of β-sheet type of structure is evident. The more completely unfolded structure displays extended parts with α-helical character (more than 50%). The qualitative agreement between increased denaturing conditions in both experiment and theory is good; an increase in the helical secondary structure content inferred from the experimental CD is recovered from the more extended structure. Hence, the solution-phase simulations are able to recover the expected transition from the native to A-state of ubiquitin.

Chromophore-tagged configurations were generated by mutating the G35 position to a donor functionalized cysteine residue, and the L73 position to the acceptor functionalized cysteine residue in both N and A states. For the native state, the crystal structure was taken as a starting point, and all amino acids except arginine are assumed to be neutral (see Supplementary Table S1), giving a final charge state of 6+ (including the charges on the two chromophores). For modeling of the A-state, the residue mutations were performed on the partially unfolded structure generated above (which now possesses 14 charges because of the chromophores, see Supplementary Table S1) and was again equilibrated in water/methanol to account for structural response to the tagging (the increase in the charge state results in further unfolding with more helical content, similar to the structure obtained after the long 400 K run).

During the 10 ns gas-phase MD runs, the initial structures of the N- and A-state models are mainly preserved, and the final structures, termed “*native*” gas-phase structures, are shown in Figure 3c. A detailed description of the theoretical determination of FRET efficiencies can be found elsewhere, and here they are estimated for these final structures using Förster theory and assuming isotropic chromophore orientations [15]. The predicted range of FRET efficiencies for the 6+ N-state model is 0.66 and 0.91 (the range is due to existence of a protruding chromophore configuration with 3.6 nm separation and a tight-fitted chromophore configuration with 2.7 nm separation, see Supplementary Figure S5). These two different structures indicate that the long linker chain of the chromophores provides enough flexibility for them to extend upon addition of protons to the protein, rather than greatly perturbing the native structure of the protein, consistent with the ion mobility data described above. The estimation of the FRET efficiency for the gas-phase A-state model (13+ charge state) amounts to less than 0.1 (chromophore separations range from 8.7 to 11.3 nm). Clearly, charge states between 6+ and 13+ would support a distribution of structures intermediate between both of these values (either by a mixture of folded and extended configurations as well as partially unfolded structures).

Finally, the effect of gas-phase relaxation of the structures to their global minimum on long timescales (and the impact on the FRET efficiencies) is considered. Replica-exchange MD simulations were performed that are able to overcome free-energy barriers and thus enable the relaxation from kinetically trapped solution-phase structures towards their gas-phase structures (termed *“gas-phase relaxed*” in Figure 3c). While the 13+ charge state does not undergo major structural changes between the chromophores (notice the transition of the remaining N-terminal β-sheet to α-helix), the 6+ structure clearly undergoes major changes in secondary structure content. More importantly, despite this structural change, the 3D arrangement of the domains is reminiscent of the native state (for example the close contact of the blue-colored N-terminal residues and C-terminal orange-red-colored residues in *native* and *gas-phase relaxed* structures of the 6+ charge state, Figure 3c). This reminiscence is reflected in the chromophore separations adopted in these structures as well. The range of sampled FRET efficiencies for the 6+ charge state becomes broader (0.96 to 0.37 with chromophore separations ranging from 2.4 to 4.4 nm). This change is much less pronounced compared with that resulting from the partial unfolding of the protein (as seen by zero FRET efficiencies in our highly charged models).

### FRET Efficiency Determination

**Figure 4:**
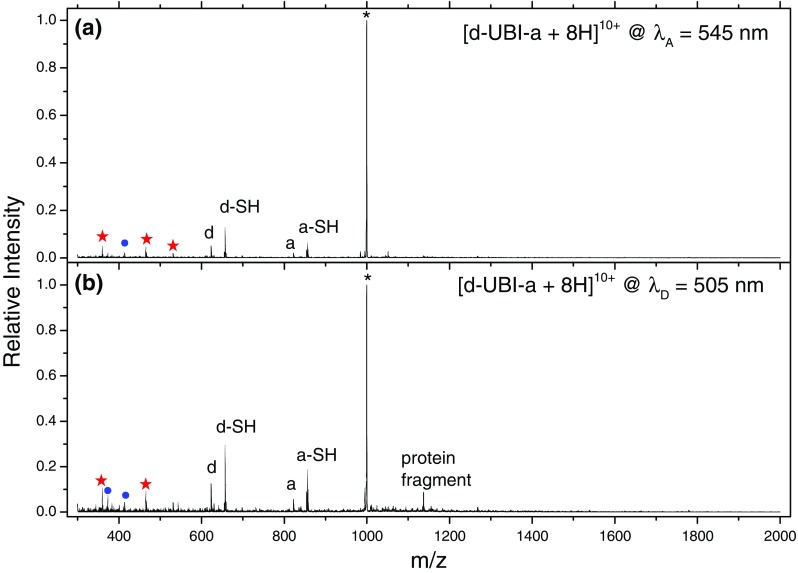
Mass spectra of mass-selected [d-UBI-a + 8H]^10+^ ions – denoted by the asterisk – following irradiation at λ_A_ = 545 nm **(a)** and λ_D_ = 505 nm **(b)**. The peaks denoted d/d-SH and a/a-SH correspond to breaking of the either side of the sulphur atom in the thioether linker. The red stars represent the acceptor chromophore-specific photofragments. The blue circles denote fragments associated with the donor chromophore

Action-FRET is dependent on the observation of photofragments specific to the acceptor chromophore when exciting the donor chromophore (in direct analogy to fluorescence-based techniques where the fluorescence of the acceptor chromophore when exciting the donor chromophore is used as a reporter of the FRET efficiency). It has been previously shown that the action-FRET efficiency can be measured by comparison of the acceptor-specific photofragmentation following excitation at 545 and 505 nm (corresponding to acceptor and donor gas-phase absorption maxima). Here, we seek to formalize the methodology for obtaining FRET efficiencies by action-FRET in order to provide a coherent method that may be easily reproduced. Figure 4 shows the mass spectrum following laser irradiation of [d-UBI-a + 8H]^10+^ (denoted by an asterisk) at Figure 4a) 545 nm and (Figure 4b) 505 nm. The photo-fragments that are specific to acceptor electronic excitation (at *m/z* 360 and 465) are denoted with red stars in the two panels. In addition to this, several other peaks are clearly visible in the mass spectrum, as detailed in Table 1. The peaks at *m/z* 623 and 657 correspond to donor and donor-SH respectively (Figure 4d and d-SH), and similarly the peaks at *m/z* 822 and 856 to acceptor and acceptor-SH (Figure 4a and a-SH). These cleavages are commonly observed in thioether-linked chromophores where the tagging has been performed by maleimide chemistry, both by collision and laser induced dissociation. In addition, peaks at *m/z* 414 and 373 (blue circles in Figure 4) observed in Figure 4b can be assigned as internal fragmentation of the donor chromophore [12]. Since fragmentation of the donor has been shown to proceed via higher lying singlet states, this may be indicative of absorption of two photons by the donor due to high laser fluence used [31]. Finally, a multiple charged peak is observed at *m/z* 1137, and the profile of which indicates it is multiply charged. The origin of this fragment is unknown, but it is interesting to note that the same *m/z* is observed for other charge states of d-UBI-a. This may indicate that this fragment is a cleavage product formed by relaxation following loss of a chromophore from one of the cysteine residues.

**Table 1:** Major Fragment Peaks Observed in the LID Mass Spectrum of [d-UBI-a +8H]^10+^ Following Laser Irradiation at Either 545 or 505 nm

*m/z*	Assignment
1110	Parent
1137	Protein fragment around cysteine?
1105	CO_2_ loss
856	QSY7-SH
822	QSY7
657	R575-SH
623	R575
531	QSY7 internal fragment
465	QSY7 internal fragment
414	R575 internal fragment
360	QSY7 internal fragment

To calculate the FRET efficiency from this data, first the acceptor specific fragmentation yield (Y_ASF_) is defined as the relative intensity of the *m/z* 360 and 465 peaks (I_ASF_) compared with the total intensity (I_total_), normalized to laser fluence (∅), i.e., *Y*_*ASF*_ = (*I*_*ASF*_/*I*_*total*_)/*ϕ*. The measurements at 545 and 505 nm are then repeated at least five times to account for changes in laser alignment and sample preparation (see Supplementary Figure S6). The values for Y_ASF_ at both wavelengths are then averaged to give a single value and the standard deviation determined. The FRET efficiency is then calculated as defined in previous studies; the acceptor specific fragmentation yield at 505 nm is normalized to the value at 545 nm (i.e., *Y*_*ASF*_^505 *nm*^/*Y*_*ASF*_^545 *nm*^). The errors are determined by propagation of the standard deviations of Y_ASF_ at λ = 545 and 505 nm.

Finally, a correction factor of −0.25 is applied to the average FRET efficiency to take into account the constant off-set in FRET efficiency attributable to the non-zero absorption of the acceptor at 505 nm. This gives values of the FRET efficiency that lie between 1 and 0, as expected.

### FRET Efficiency Versus Charge

The conformational ensemble that is sampled depends not only upon the solution conditions used for ESI but also on the charge state. As noted above, folded proteins tend to acquire less charge upon ESI than do extended structures of the same protein. This will not only affect the charge distribution as shown in Figure 2 but also determine the conformational ensemble that is sampled by a particular charge state, as shown in Supplementary Figures S2 and S3. Therefore, the sampled conformational ensemble of each charge state is determined by both the solution-phase conformational ensemble and by the charge distribution of folded and extended structures acquired during the ESI process. It follows that low charge states will have conformational ensembles derived from a high proportion of folded structures, high charge states containing a high proportion of extended structures, and the intermediate charge states containing a mixture of the two. Ultimately, it is possible to use information about the intensity of a particular charge state and its conformational ensemble to reconstruct the total ensemble [19].

**Figure 5:**
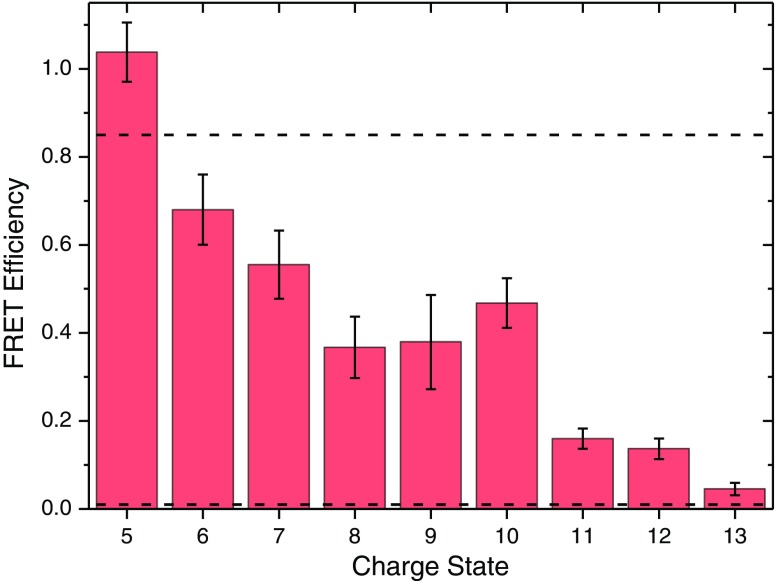
FRET efficiency as a function of the charge state of [d-UBI-a + (z-2)H]^z+^ cations produced by ESI from a 1:1 solution of H_2_O:CH_3_OH with 1% acetic acid by volume. The dashed lines indicate estimated FRET efficiencies for the native (N) and A-states of doubly grafted ubiquitin

Figure 5 shows the FRET efficiencies for [d-UBI-a + (z-2)H]^z+^ (where z = 5–13 is the total charge state) following ESI from 1:1 H_2_O:CH_3_OH with 1% acetic acid by volume as solid bars (see Table 2). There are three clear steps in the FRET efficiency between z = 5 and z = 6, between z = 7 and z = 8, and between z = 10 and z =11. Considering the drop between z = 10 and z = 11 first, the CCS data in Supplementary Figure S2 shows that there is a change from predominantly folded to predominantly unfolded between z = 7 and z = 8 for C-UBI-C, which is consistent with the CCS values of Wyttenbach and Bowers for bovine ubiquitin [21]. For a-UBI-d the transition is observed between the z = 8 and z = 10, which is consistent with the FRET data in Figure 5. We can therefore assign this change in FRET efficiency to a change in conformational ensemble from predominantly folded to predominantly unfolded.

**Table 2:** FRET Efficiency Values in 1:1 H_2_O:CH_3_OH with 1% Acetic Acid by Volume

	FRET efficiency
[d-UBI-a +3H]^5+^	1.04 ± 0.08
[d-UBI-a +4H]^6+^	0.68 ± 0.10
[d-UBI-a +5H]^7+^	0.56 ± 0.10
[d-UBI-a +6H]^8+^	0.37 ± 0.11
[d-UBI-a +7H]^9+^	0.39 ± 0.10
[d-UBI-a +8H]^10+^	0.41 ± 0.09
[d-UBI-a +9H]^11+^	0.16 ± 0.05
[d-UBI-a +10H]^12+^	0.14 ± 0.06
[d-UBI-a +11H]^13+^	0.06 ± 0.07

Next, the decrease in FRET efficiency between z = 5 and z = 6 is considered. As was discussed above, two different structural families with a native protein fold were found differing only in the extension of the donor chromophore linker chain. The experimental value (0.68 ± 0.1) for [d-UBI-a + 4H]^6+^ and is in very good agreement with the value for structure with donor linker chain extended (0.66), found in the molecular dynamics simulations. Similarly, the close-fitting native structure (neither linker chain is extended) FRET efficiency (0.91) is close to the experimental value of 1.04 ± 0.08 for [d-UBI-a + 3H]^5+^. It is therefore possible that the initial decrease in the FRET efficiency observed for z = 5 and z = 6 is due to the extension of the donor linker chain, all within the native fold of ubiquitin. Likewise, it is possible to hypothesize that the decrease observed between z = 7 and z = 8 is a transition to a structure where both chromophore linker chains are extended, a structure not sampled in the 6+ model. In this case, the small decrease would represent a distribution of structures with either one or both chromophores extended away from the protein. Alternatively, it could be that a greater proportion of the sampled conformational ensemble is in an unfolded, non-native state where the chromophore separation is high and hence FRET efficiency is low. However, the plateau between z = 8 and z = 10 is indicative of a stable conformational ensemble, which is in favor of the former explanation.

### FRET Efficiency Versus Electrospray Solvent

**Figure 6:**
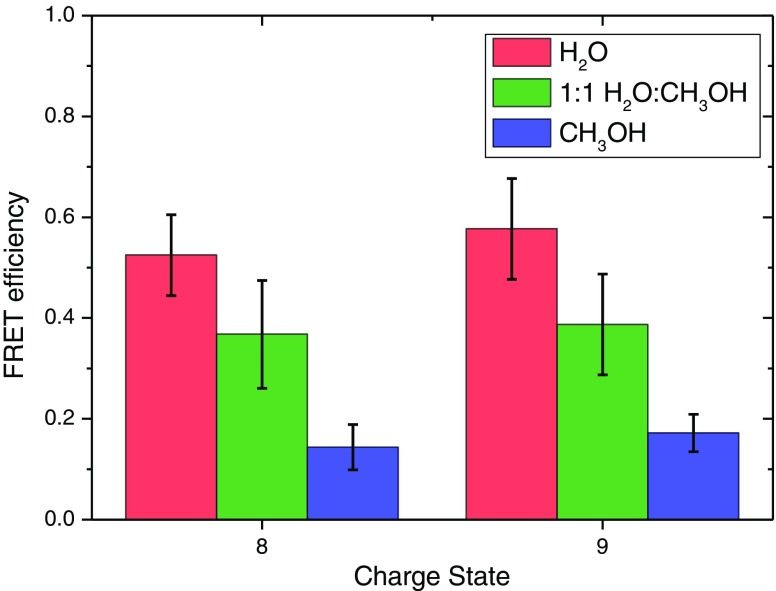
FRET efficiencies of [d-UBI-a + 6H]^8+^ and [d-UBI-a + 7H]^9+^ following ESI from either H_2_O (red), 1:1 H_2_O:CH_3_OH (green) or CH_3_OH (blue) solutions with 1% acetic acid by volume

The relationship between the conformational ensemble in solution and gas phases is one of the most important open questions relating to the use of native mass spectrometry in the scope of structural biology. One of the motivations for the development of gas-phase FRET techniques is to have a direct connection between solution- and gas phases. An important first step is, therefore, to show that action-FRET is indeed sensitive to the electrospray solvent used, in order to demonstrate that there is some memory of the solution-phase conformational ensemble upon its transposition to the gas phase. Figure 6 shows the FRET efficiencies for the z = 8 and z = 9 charge states of [d-UBI-a] following ESI from either H_2_O, 1:1 H_2_O:CH_3_OH, or CH_3_OH, each with 1% acetic acid by volume. It should be noted that in the pure methanol case, there is ~5% by volume H_2_O remaining because of the initial reaction solution being in water. In order to ensure that it was the ESI solvent causing any differences, a fresh aliquot of tagged ubiquitin was prepared, and the subsequent dilution into each solution and experiments were performed sequentially.

There is a clear decrease in FRET efficiency as the methanol concentration of the ESI solvent is increased. This indicates that the conformational ensembles sampled by both protonation states contain more compact structures in H_2_O, and more extended structures in CH_3_OH, which is consistent with the denaturing properties of the latter medium. This strongly indicates that there is a memory of the solution-phase conformational ensembles in the experimentally probed gas-phase ensemble after denaturation in acidified methanol, as has been previously noted by Shi et al. [19].

It is equally important to show that the changes in gas-phase conformational ensembles as a function of electrospray solvent as measured by action-FRET are consistent with the changes in the observed solution-phase conformation ensembles under the same solution conditions. The circular dichroism as a function of the methanol concentration under identical solution conditions to those used in the electrospray are shown for bovine ubiquitin in Supplementary Figure S4. There is a small change in the spectrum comparing H_2_O and the binary methanol water mixture, and a much larger change for the methanol only solution. As was noted above, these changes correlate to the degree of the unfolded A-state, which presents an increasingly helical character as the conditions are made more denaturing.

It is possible to estimate the amount of α-helical and β-strand character present in each sample using the K2D3 prediction software [56], the results of which are shown in Supplementary Table S1, alongside the predicted values for the reference spectrum shown in Figure 2. There is a clear increase in the α-helix content of the protein as the concentration of methanol is increased, with a concurrent decrease in the β-strand content. This is consistent with the expected change from native structure in H_2_O, which possesses a high percentage of β-strand secondary structure, to the predominantly helical A-state present in methanol. It is also consistent with NMR data, which show that α-helix secondary structure content is vastly increased in 30% methanol solutions compared with pure water [44]. It is therefore possible to conclude that the change in the FRET efficiencies is indeed due to the memory retention of the solution-phase conformational ensemble sampled in different denaturing conditions. These experimental data in both solution and gas phase are consistent with the FRET efficiencies, and although they do not yet allow a direct comparison of the solution and gas-phase conformational ensembles, they show how such an experiment is possible.

## Conclusions and Prospects

In this paper, a clear methodology for performing action-FRET measurements in the gas phase has been presented from identification of the mutation sites on a target protein to the data analysis and interpretation of FRET efficiencies. A G35C L73C mutant of ubiquitin was produced where the tagging locations are expected to undergo a large change in separation when ubiquitin goes from the folded native state to the unfolded A-state. It was demonstrated that the tagging of this mutant can be performed with close to 100% efficiency, although the nonspecific nature of the grafting reduces the quantity of protein tagged with a single donor and acceptor. Measurement of circular dichroism, charge state distributions, and collision cross sections was used to probe the influence of the mutation and tagging protocol on the protein structure. It was concluded that there is no large change in secondary structure due to the two point mutations, and that although there is some evidence for destabilization by the presence of the charged chromophores, protein unfolding is predominantly driven by the protonation state of the protein. A coherent method was presented for performing action-FRET measurements in a repeatable manner. Several of the factors that may affect the absolute value of the FRET efficiency have also been discussed, with direct absorption and subsequent fragmentation of the acceptor chromophore at the donor absorption maximum corrected.

The FRET efficiency of tagged ubiquitin was considered both as a function of the charge state and the electrospray solvent. It was shown that the results of ensemble action-FRET measurements were completely consistent with previous measurements of the conformational ensembles of ubiquitin by ion-mobility spectrometry, and that this data can be interpreted by consideration of both the solution-phase structural ensemble and the manner of charging during ESI. Molecular dynamics simulations showed that the trend of FRET efficiency as a function of charge state can be understood by both local changes in chromophore orientation on a fixed protein structure and changes in the protein secondary structures.

It was also shown that changes in the FRET efficiency for a fixed charge state could be observed as the methanol concentration of the electrospray solvent was changed. This provides further evidence for memory of the solution-phase conformational ensembles upon their transposition to the gas phase. It also hints that action-FRET—and gas phase FRET techniques in general—may be able to provide a direct link between solution- and gas-phase experiments.

## Electronic supplementary material

Below is the link to the electronic supplementary material.

ESM 1 (DOCX 236 kb)
