# Supplementary material for: Action-FRET of a Gaseous Protein
Source: J Am Soc Mass Spectrom. 2016 Aug 9;28(1):38–49. doi: 10.1007/s13361-016-1449-2 (PMC5174150; doi:10.1007/s13361-016-1449-2)
Supplement: Supplementary file 1 — (DOCX 236 kb) [file 13361_2016_1449_MOESM1_ESM.docx]

Supplementary Information.

Action-FRET of a gaseous protein.

S. Daly^a^, G. Knight^a^, M.A. Halim^a^, A. Kulesza^a^, Chang Min Choi^a^, L. MacAleese^a^, R. Antoine^a^, and P. Dugourd^a*^.

^a^. Institut Lumière Matière, Université Lyon 1-CNRS, Université de Lyon 69622 Villeurbanne cedex, France.

* Corresponding author email: philippe.dugourd@univ-lyon1.fr

| Residue | Untagged 12+ | | Tagged 6+ | | Tagged 14+ | |
| --- | --- | --- | --- | --- | --- | --- |
|  | pdb code | charge | pdb code | charge | pdb code | charge |
| K6 | LYS | +1 | LYN | 0 | LYS | +1 |
| K11 | LYS | +1 | LYN | 0 | LYS | +1 |
| E16 | GLH | 0 | GLH | 0 | GLH | 0 |
| E18 | GLH | 0 | GLH | 0 | GLH | 0 |
| D21 | ASH | 0 | ASH | 0 | ASH | 0 |
| E24 | GLH | 0 | GLH | 0 | GLH | 0 |
| K27 | LYS | +1 | LYN | 0 | LYS | +1 |
| K29 | LYS | +1 | LYN | 0 | LYS | +1 |
| D32 | ASH | 0 | ASH | 0 | ASH | 0 |
| K33 | LYS | +1 | LYN | 0 | LYS | +1 |
| D34 | ASH | 0 | ASH | 0 | ASH | 0 |
| D39 | ASH | 0 | ASH | 0 | ASH | 0 |
| R42 | ARG | +1 | ARG | +1 | ARG | +1 |
| K48 | LYS | +1 | LYN | 0 | LYS | +1 |
| E51 | GLH | 0 | GLH | 0 | GLH | 0 |
| D52 | ASH | 0 | ASH | 0 | ASH | 0 |
| R54 | ARG | +1 | ARG | +1 | ARG | +1 |
| D58 | ASH | 0 | ASH | 0 | ASH | 0 |
| K63 | LYS | +1 | LYN | 0 | LYS | +1 |
| E64 | GLH | 0 | GLH | 0 | GLH | 0 |
| H68 | HIP | +1 | HIS | 0 | HIP | +1 |
| R72 | ARG | +1 | ARG | +1 | ARG | +1 |
| R74 | ARG | +1 | ARG | +1 | ARG | +1 |

**Table S1.** Peptide codes and nominal charge of all amino acids with charged side chains used in molecular dynamics solutions. Note that in the tagged species, each chromophore also carries +1 charge.

| Structure | α helix Content % | β strand content % |
| --- | --- | --- |
| 1UBQ^a^ | 16 | 30 |
| A1^b^ | 10 | 14 |
| A2^c^ | 50 | 0 |

**Table S2.** Estimation of the secondary structure using the STRIDE program [1]. ^a^ crystal structure taken from pdb file 1UBQ. ^b^ 37.5 ns 300K partially unfolded run (sampled using 1500 structures) 0-28 % alpha (10 % average) 8-17% beta (14 % average). ^c^ A2 -- unfolded (structure after 75 ns @400K): 50 % alpha, 0% bet

| Solution conditions | α helix Content % | β strand content % |
| --- | --- | --- |
| H_2_O | 4 | 33 |
| H_2_O:CH_3_OH | 19 | 25 |
| CH_3_OH | 54 | 5 |
| Reference^a^ | 12 | 28 |

**Table S3.** Estimation of the secondary structure content from the circular dichroism spectra in different solution conditions using the K2D3 software prediction tool. ^a^ taken from reference[48, 49].

[1] D. Frishman, P. Argos, *Proteins Struct. Funct. Genet.* **1995**, *23*, 566–579.

**Figure S1**. Circular dichroism signal for the G35C L73C ubiquitin mutant (solid black curves, left axis) and wild-type bovine ubiquitin (solid blue curves, right axis) in H_2_O with 1% acetic acid by volume. A reference spectrum taken from reference [48, 49] is shown as the dashed red curve.

**Figure S2.** Collision cross section values (in He) of the highest peak for untagged C-UBI-C (black squares) and acceptor doubly tagged ubiquitin a-UBI-a (red circles) as a function of the charge state.

**Figure S3.** Collision cross section profiles (in He) for untagged mutant ubiquitin (left) and acceptor doubly tagged ubiquitin (right) where z is the total charge state of the system.

**Figure S4.** (a) Circular dichroism spectra for wild-type bovine ubiquitin in H_2_O (black), 1:1 H_2_O:CH_3_OH (red) and CH_3_OH (blue), each with 1% acetic acid by volume. (b) Predicted circular dichroism spectra for the ubiquitin crystal structure (pdb file 1UBQ), and the A1 and A2 unfolded structures found in in solution-phase molecular dynamics simulations.


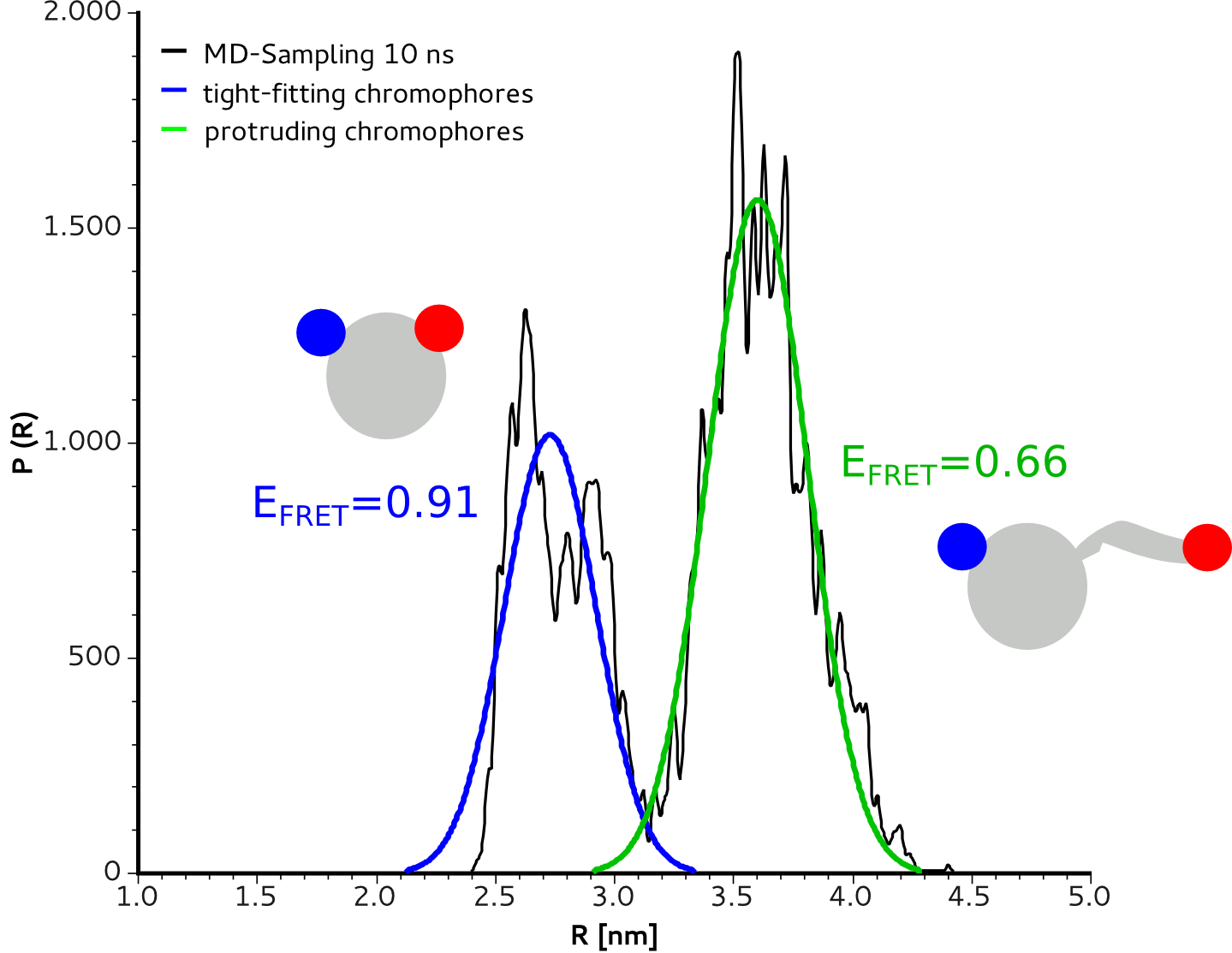


**Figure S5.** Calculated chromophore-distance distributions of the chromophore-tagged ubiquitin in the 6+ charge state. Structural samples comprise two 37.5 ns runs of the partially unfolded structure after tagging at 300 K (both chromophore-tagging configurations with Rh575 at position 35 and 73, respectively). The obtained probability density P(R) (black line) was fitted with two Gaussians at corresponding to tight-fitted (blue, R centred at 2.73 nm) and protruding chromophores (green, R centred at 3.6), both with intact native fold of the protein (notice that the length of one C5-maleimide linker chain is about 0.9 nm). The two chromophore configurations of the native state correspond to FRET efficiencies of 0.91 (tight-fitted) and 0.66 (protruding), averaging to 0.78 for the mixture. Thus, native state FRET-efficiencies are expected to tune from nearly 1 to 0.66 by charge state without major disturbance of the native state protein structure.

Figure S6. Experimental FRET efficiency for 6 data sets of mass selected [a-UBI-d + 8H]^10+^ Repeats 1-5 were taken on the same day under identical conditions (including sample).Repeat number 6 was performed 6 days later as a single measurement from a different sample. Here we can nicely demonstrate that the FRET efficiencies are reproducible both within the same sample and experimental setup; and with different sample and laser realignment.
